# Protective effect of anisodamine in rats with glycerol-induced acute kidney injury

**DOI:** 10.1186/s12882-019-1394-y

**Published:** 2019-06-17

**Authors:** Yun-feng Li, Bing-yuan Xu, Ran An, Xin-fang Du, Kun Yu, Jia-hua Sun, Guo-hong Zhang, Wei Wang, Li-ping An, Guang-li Wu

**Affiliations:** 1 Hebei Key Laboratory of Chinese Medicine Research on Cardiocerebrovascular Disease, Hebei University of Chinese Medicine, Shijiazhuang, 050200 China; 2 Department of Nephrology, Bethune International Peace Hospital of PLA, Shijiazhuang, 050082 China

**Keywords:** Anisodamine, Atropine, Acute kidney injury, Rhabdomyolysis

## Abstract

**Background:**

Anisodamine is used for the treatment of reperfusion injury in various organs. In this study, we investigated the effectiveness and mechanisms of action of anisodamine in promoting recovery from glycerol-induced acute kidney injury (AKI).

**Methods:**

We compared the protective effects of atropine and anisodamine in the rat model of glycerol-induced AKI. We examined signaling pathways involved in oxidative stress, inflammation and apoptosis, as well as expression of kidney injury molecule-1 (KIM-1). Renal injury was assessed by measuring serum creatinine and urea, and by histologic analysis. Rhabdomyolysis was evaluated by measuring creatine kinase levels, and oxidative stress was assessed by measuring malondialdehyde (MDA) and superoxide dismutase (SOD) levels in kidney tissues. Inflammation was assessed by quantifying interleukin 6 (IL-6) and CD45 expression. Apoptosis and necrosis were evaluated by measuring caspase-3 (including cleaved caspase 3) and RIP3 levels, respectively.

**Results:**

Glycerol administration resulted in a higher mean histologic damage score, as well as increases in serum creatinine, urea, creatine kinase, reactive oxygen species (ROS), MDA, IL-6, caspase-3 and KIM-1 levels. Furthermore, glycerol reduced kidney tissue SOD activity. All of these markers were significantly improved by anisodamine and atropine. However, the mean histologic damage score and levels of urea, serum creatinine, creatine kinase, ROS and IL-6 were lower in the anisodamine treatment group compared with the atropine treatment group.

**Conclusion:**

Pretreatment with anisodamine ameliorates renal dysfunction in the rat model of glycerol-induced rhabdomyolytic kidney injury by reducing oxidative stress, the inflammatory response and cell death.

## Background

Acute kidney injury (AKI) is a serious disease with a high mortality rate. Rhabdomyolysis is a common clinical disorder with a broad spectrum of traumatic and non-traumatic etiologies, and approximately 10 to 50% of patients suffering from rhabdomyolysis develop some degree of AKI [1, 2]. Renal tubular damage is a pathological characteristic of AKI. Currently, animal models of glycerol-induced AKI are widely used [3]. Glycerol injection into the muscle causes the release of myoglobin and other muscle contents into the circulation, ultimately resulting in AKI. Recent studies have demonstrated that the pathogenesis of glycerol-induced AKI involves myoglobin toxicity [4–6], reactive oxygen species (ROS) [7–9], inflammation [10], apoptosis [11, 12] and redox-active iron [7]. Although the pathogenesis of glycerol-induced AKI is complex, timely prophylactic and/or early therapeutic interventions can promote recovery [8, 12, 13]. Anisodamine, derived from *Scopolia tangutica* Maxim, is used for the treatment of gastrointestinal smooth muscle spasm, infective toxic shock, myocardial infarction and acute lung injury in China [14–17]. Anisodamine and atropine are non-specific cholinergic antagonists with the usual spectrum of pharmacological effects typical of this drug class. However, anisodamine appears to be less potent and less toxic than atropine, which is widely used in clinical and basic research [18].

Anisodamine has been shown to be effective in improving the microcirculation of the hydronephrotic kidney in the rat [19]. No published report has examined the efficacy of delayed therapeutic intervention when renal dysfunction is already well established. In our previous study (data not published), anisodamine was effective in the treatment of AKI. However, the mechanisms by which anisodamine promotes recovery from renal dysfunction in the rat AKI model remain unclear, although they may involve the inhibition of apoptosis and the suppression of inflammatory cytokine production.

In this study, we used the rat glycerol-induced acute renal injury model to clarify the mechanisms underlying the therapeutic effectiveness of anisodamine. We investigated the effects of the delayed administration of anisodamine on renal function and pathology by examining biomarkers of AKI. Our findings suggest that anisodamine improves renal function by affecting leukocyte infiltration and inflammation, oxidative stress and apoptosis.

## Materials and methods

### Animal groups, randomisation and tissue collection

Male Sprague-Dawley rats at 8 weeks of age (190–210 g) were purchased from Hebei Medical University and housed in metabolic cages under standard conditions, with food and water available ad libitum, in a room with a 12/12-h light/dark cycle (lights on from 08:00 to 20:00 h) and controlled temperature (21 ± 1 °C). All procedures involving animals were conducted in accordance with the National Institutes of Health Guide for the Care and Use of Laboratory Animals and were approved by the Animal Ethics and Use Committee of Hebei Science and Technical Bureau in the People’s Republic of China.

The block randomisation scheme will be generated by a computer-generated random assignment sequence prepared in advance. First, the rats were labeled with codes of Arabic numerals in same cage (same genetic background). In each cage, there will be labeled numerically with these codes, then the labeled codes were inputted into computer. An independent statistician who is not directly participant in the conduct of the trial will generate the randomisation sequence with computer.

The rats were fasted (food and water) for 24 h before glycerol injection, and then divided randomly into nine groups (see Table 1) according to trial design with block randomization. Group 1 (*n* = 5) was not given any treatment. Groups 2–5 (*n* = 45) were given intramuscular injections of 50% glycerol (10 mL/kg) in their hind limbs. Groups 1 and 2 received sterile water, while group 3 received anisodamine (Raceanisodamine Hydrochloride Injection, Hangzhou Minsheng Pharmaceutical Group Co., Ltd.) by intraperitoneal injection (1 mg/kg) 20 min before the initial glycerol injection. Groups 4 and 5 each received atropine (atropine sulfate injection, Hangzhou Minsheng Pharmaceutical Group Co., Ltd.) by intraperitoneal injection (0.05 mg/kg and 2 mg/kg) 20 min before the initial glycerol injection. Groups 6–9 (*n* = 37) were given intramuscular injections of 50% glycerol (15 mL/kg) in their hind limbs. Group 6 received sterile water, while group 7 was given anisodamine by intraperitoneal injection (1 mg/kg) 20 min before the initial glycerol injection. Groups 8 and 9 each received atropine by intraperitoneal injection (0.05 mg/kg and 2 mg/kg) 20 min before the initial glycerol injection. Rats were placed in metabolic cages for 24-h urine collections. The animals were euthanized with 10% chloral hydrate (4.5 ml/kg). Blood and urine were collected at different time points for estimation of serum creatinine, blood urea nitrogen and creatine kinase. The kidneys were harvested (*n* ≥ 3 at each time point) for further analysis. Part of each kidney was fixed in 4% paraformaldehyde solution. The remaining tissue was frozen immediately in liquid nitrogen and stored at − 80 °C.

**Table 1:** Group experiment

	1	2	3	4	5	6	7	8	9
Glycerol (10 mL/kg)		√	√	√	√				
Glycerol (10 mL/kg)						√	√	√	√
Sterile water	√	√				√			
Anisodamine (1 mg/kg)			√				√		
Atropine (0.05 mg/kg)				√				√	
Atropine (2 mg/kg)					√				√

### Sample size calculation and inclusion/exclusion criteria

This study is designed primarily to explore the mechanism of protective effect of anisodamine on glycerol-induced acute kidney injury in rats. We will aim to collect experimental data as many rats as possible according to common animal experiment design groups (6–9 rats/ each group). Eighty-seven rats are divided nine groups, which would be able to give 95% confidence. The data from this animal experiment will be used to refine sample size calculations for future randomized controlled trial.

Rat are eligible for inclusion if they are as follows:

- Weight between 190 g and 210 g
- Male rats
- Survival rats after treatment

Exclusion criteria

Rats are excluded if they have one or more of the following:

- The weight less than 150 g or greater than 280 g
- The dead rats after treatment
- Female rats

### Assessment of renal function

Renal function was monitored by measuring serum creatinine (Cat. no. C011–2), blood urea nitrogen (Cat. no. C103–2) and creatine kinase (Cat. no. A032) using assay kits (Nanjing Jiancheng Bioengineering Institute, Jiang Su, China) according to the manufacturer’s instructions.

### Kidney histology

Kidney tissues were fixed in 4% paraformaldehyde and routinely processed for paraffin embedding. Sections were stained with hematoxylin and eosin for histological assessment, and images were obtained with an Olympus DP70 digital camera (Olympus Optical Co, Ltd., Tokyo, Japan) and analyzed with Image-Pro Plus 6.0 Software (Media Cybernetics, Inc., Bethesda, MD, USA). The changes were limited to the tubulointerstitial areas, and were graded as follows (described previously in [20, 21]): (I) areas of tubular epithelial cell swelling, vacuolar degeneration, necrosis and desquamation involving < 25% of cortical tubules; (II) similar changes involving > 25% but < 50% of cortical tubules; (III) similar changes involving > 50% but < 75% of cortical tubules; (IV) similar changes involving > 75% of cortical tubules.

### Immunohistochemistry and evaluation of immunostaining

All incubations were carried out at room temperature, unless otherwise stated. Immunohistochemistry was conducted for KIM-1 (ab78494, Abcam), caspase-3 (#9662, Cell Signaling Technology), cleaved caspase 3 (#9664, Cell signaling Technology), RIP3 (ab62344, Abcam) and CD45 (bs-4819R, Bioss) in longitudinal sections of the kidney at the different time points. Briefly, the sections were deparaffinized and re-hydrated in water, and then immersed in citrate buffer (pH 6.0; 95 °C for 15 min) for antigen retrieval. Endogenous peroxidase activity was quenched by immersion in 3% hydrogen peroxide for 10 min. Thereafter, the sections were blocked with 5% goat serum or 1% BSA in TBS. Slides were incubated overnight at 4 °C with primary antibodies, and then with horseradish peroxidase (HRP)-conjugated secondary antibodies (Zhongshan, Beijing, China) for 30 min at room temperature. The sections were developed with 3,3′ diaminobenzidine solution. Negative control slides were treated in the same manner, but incubated with an isotype-matched non-specific immunoglobulin.

Digital images of the sections were captured and evaluated in a blind manner. All sections were evaluated for the percentage of positive cells and labeling intensity. The percentages of positive cells were assigned scores as follows: 1, < 5%; 2, 5–25%; 3, 21–50%; 4, 50–75%. The intensity was scored as follows: 0, negative staining; 1, weak staining; 2, intermediate staining; 3, strong staining. The score was calculated by multiplying the percentage of positive cells (1–4) by the staining intensity (0–3), to obtain a value of 0–12. Cells were counted under a 40× objective.

### Determination of MDA levels and SOD&IL-6 activity

Kidney tissue was gently homogenized in homogenization buffer (10 mM Tris-HCl, 1 mM EDTA, pH 7.4) and centrifuged at 5000 rpm for 10 min at 4 °C. The protein concentration in the supernatant was determined using the bicinchoninic acid assay (Nanjing Jiancheng Bioengineering Institute). The supernatant was then used for the determination of MDA levels (A003, Nanjing Jiancheng Bioengineering Institute), SOD activity (A001, Nanjing Jiancheng Bioengineering Institute) and IL-6 activity (PI328, Beyotime) using kits according to the supplier’s instructions.

### Western blotting

Protein expression levels were determined by western blot analysis as previously described [20]. In brief, the PVDF membrane was blocked with 5% w/v dried non-fat milk in Tris buffer with 0.1% Tween-20 for 1 h, and then incubated with primary antibody to KIM-1, caspase-3, RIP3, IL-6 (bs-4539R, Bioss), RIP-3 or β-actin (1:1000) (CoWin, Beijing, China) at 4 °C overnight, followed by incubation with an HRP-conjugated goat anti-rabbit secondary antibody (1:10,000). Immunoreactive bands were detected using enhanced chemiluminescence (ECL) substrate (Transgen, Beijing, China) and the Vilber Fusion FX7 system.

### Statistical analysis

The results were expressed as mean ± SEM (*n* = 4–6). A single comparison between two groups was performed with an unpaired, two-tailed Student’s *t*-test or one-way analysis of variance (ANOVA). Multiple comparisons among three or more groups were performed with an ANOVA post hoc test. *P* < 0.05 was regarded as significant.

## Results

### Observation of kidney morphology after glycerol/anisodamine/atropine treatment

**Fig. 1:**
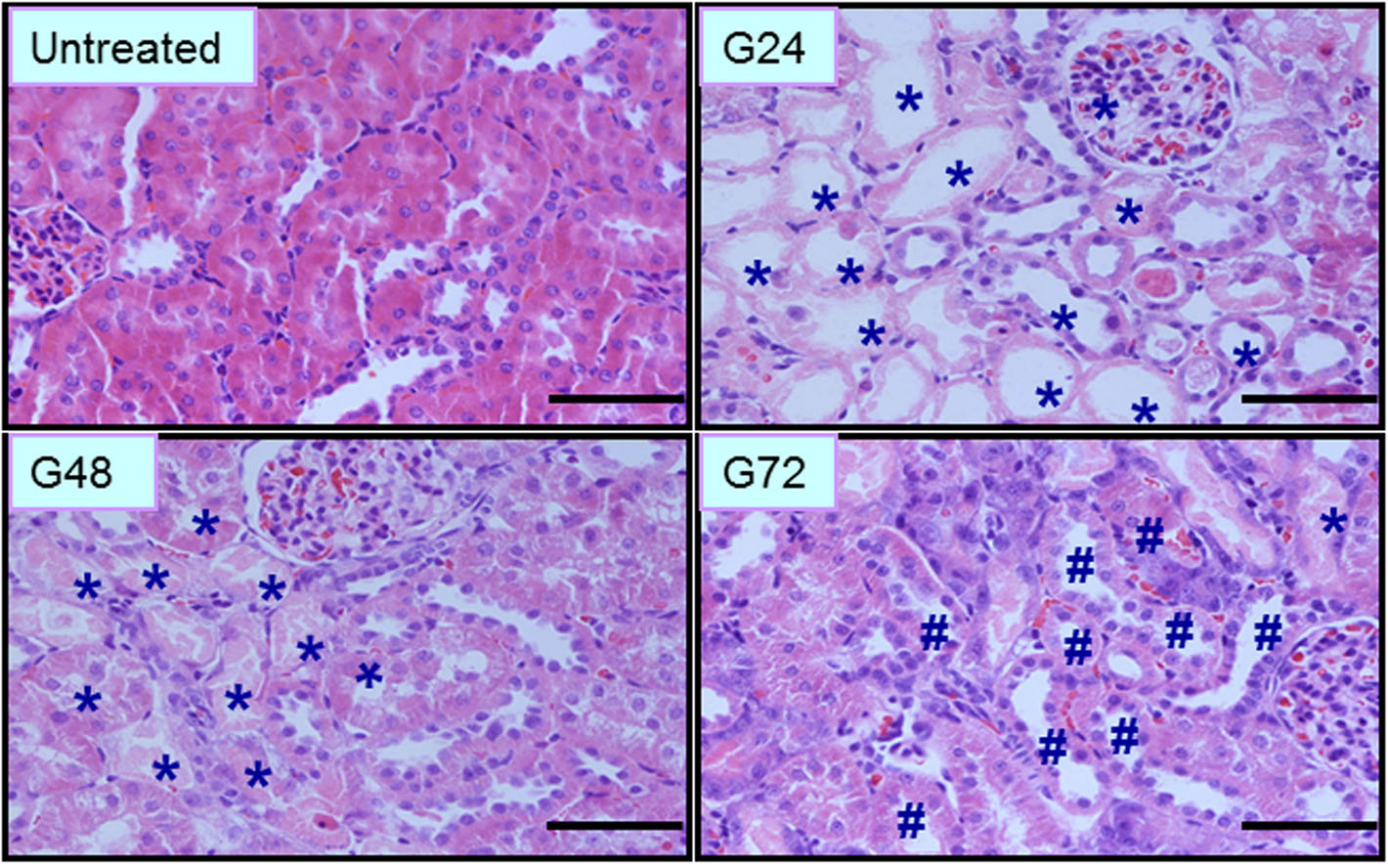
Representative morphological changes in kidney shown with H&E staining at different time points after glycerol treatment. *:desquamation and necrosis of tubular epithelial cells in the proximal and distal tubules; #: regenerative epithelial cells in the proximal and distal tubules

Histopathological analysis of kidney tissue was performed after glycerol administration. As shown in Fig. 1, at 0 h, the morphology of renal tissues was normal, with the inner part of the organ appearing soft and reddish in longitudinal sections in the control group. Pathological changes gradually increased in the AKI group from 3 to 72 h, including tubular epithelial cell swelling, vacuolar degeneration, necrosis and desquamation. At 24 h, the epithelial cells showed desquamation and necrosis, and a number of protein casts and exfoliated tubular epithelial cells were visible in the proximal and distal tubules. These changes were classified as grade IV. At 48 h, the renal capsular space widened. The epithelial cell debris appeared desquamated and disseminated into the tubular lumen. Cast formations could be also seen in the distal tubules. These changed were classified as grade III. At 72 h, the protein casts in the tubular lumens gradually dissolved and disappeared, and regenerating epithelial cells could be seen intermittently at the sites of injury in the tubules.

**Fig. 2:**
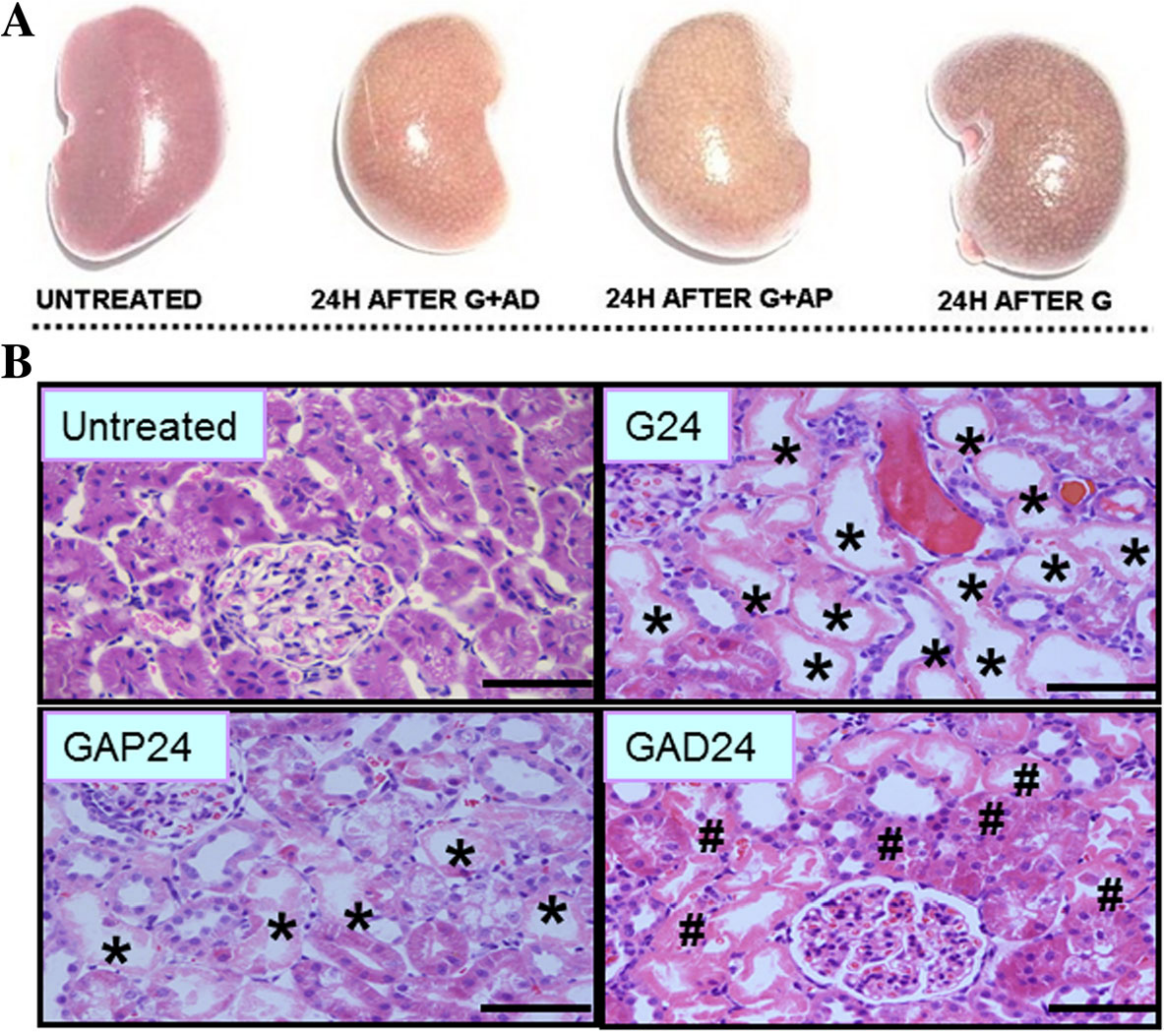
Effect of anisodamine and atropine on macroscopic and morphological changes. **a** Representative macroscopic changes in the kidney at 24 h with anisodamine and atropine administration. **b** Representative morphological changes in the kidney assessed by H&E staining at 24 h with anisodamine and atropine. Scale bar:50 μm. G, glycerol; (GAP), glycerol + atropine; (GAD): glycerol + anisodamine

We next examined gross pathological changes in the kidney 24 h after anisodamine/atropine treatment. As shown in Fig. 2a, kidneys in the untreated group were dark pink in color, which indicated that the organs were healthy. In comparison, in the glycerol treatment group, the kidneys were dark red and covered with white spots, suggesting that the organs were unhealthy. Kidneys in the anisodamine and atropine treatment group were light brown in color and sparsely covered with white spots, indicating that they were also injured. The kidney in the anisodamine treatment group appeared to have a slightly darker shade than that in the atropine group (Fig. 1a). Moreover, kidney weight and volume were slightly lower in the anisodamine treatment group than in the atropine group.

Histopathological analysis was performed 24 h after anisodamine or atropine treatment (Fig. 2b). In the anisodamine and atropine treatment groups, areas of tubular epithelial cell swelling, necrosis and desquamation were decreased compared with the control group. The degree of tissue damage was less in the anisodamine group compared with the atropine group, as indicated by smaller areas of tubular epithelial necrosis and desquamation.

### Anisodamine alleviates glycerol-induced renal dysfunction and damage

**Fig. 3:**
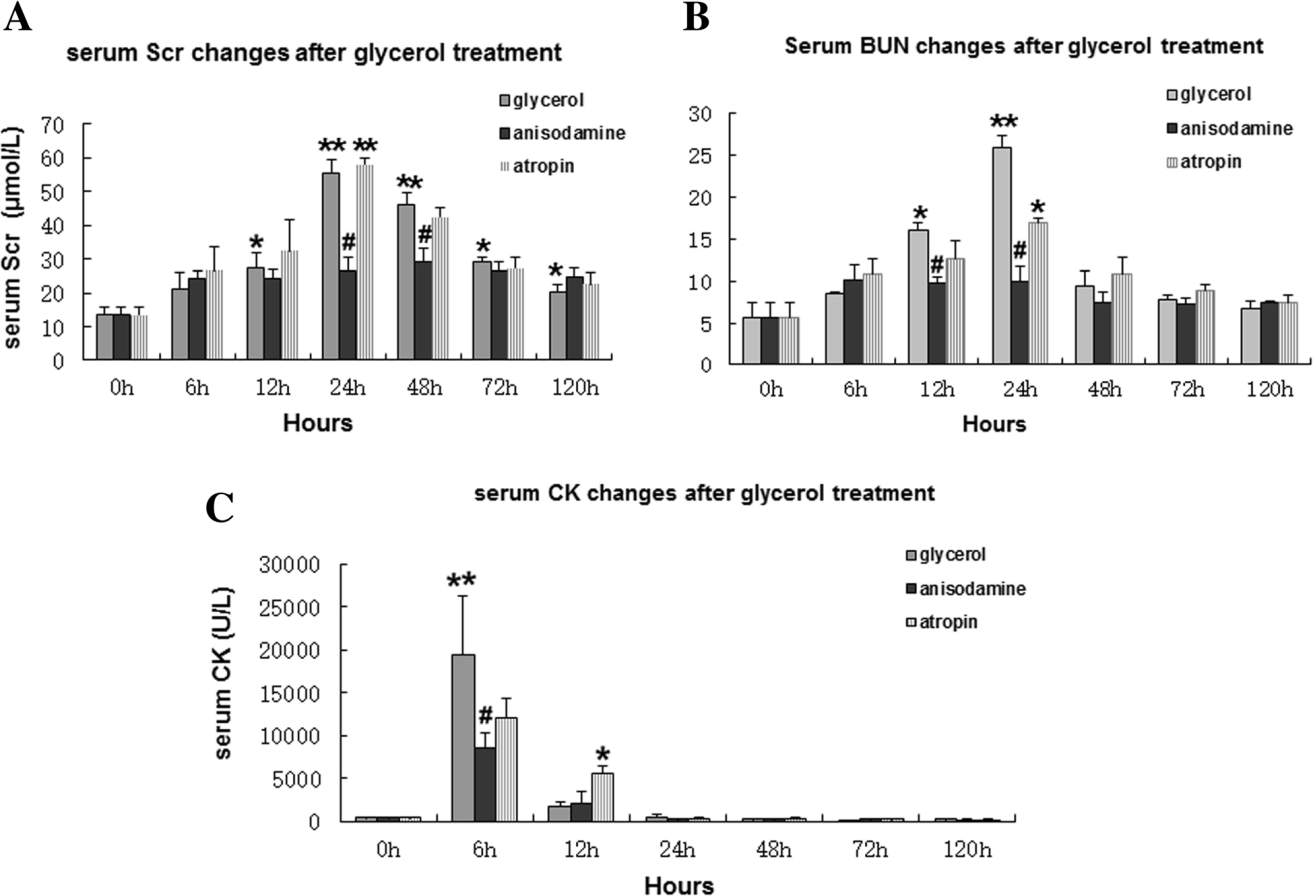
Effect of advanced administration of anisodamine/atropine on kidneys in rats subjected to glycerol-induced AKI. **a** Serum Scr in groups of male SD rats (*n* = 6) given intramuscular injections of 50% glycerol (10 mL/kg) with anisodamine (1 mg/kg) and atropine (0.05 mg/kg) by intraperitoneal injection 20 min before glycerol treatment. The control group did not receive any treatment. Data are expressed as the mean ± SEM (*n* = 6). #, statistically significantly different from respective 0 h controls; *, statistically significantly different from rats receiving glycerol treatment alone at the corresponding time point (*P* < 0.05). **b** Serum BUN in groups of male SD rats (*n* = 6) given intramuscular injections of 50% glycerol (10 mL/kg), with anisodamine (1 mg/kg) and atropine (0.05 mg/kg) by intraperitoneal injection 20 min before glycerol treatment. The control group did not receive any treatment. Data are expressed as the mean ± SEM (*n* = 6). #, statistically significantly different from respective 0 h controls; *, statistically significantly different from rats receiving glycerol treatment alone at the corresponding time point (*P* < 0.05). **c** Serum CK in groups of male SD rats (*n* = 6) injected intramuscularly with 50% glycerol (10 mL/kg), with anisodamine (1 mg/kg) and atropine (0.05 mg/kg) by intraperitoneal injection 20 min before glycerol treatment. The control group did not receive any treatment. Data are expressed as the mean ± S.E. (*n* = 6). #, statistically significantly different from respective 0 h controls (*P* < 0.05). *, statistically significantly different from rats receiving glycerol treatment alone at the corresponding time point (*P* < 0.05)

Levels of serum creatinine, blood urea nitrogen and creatine kinase were analyzed to assess renal dysfunction and damage. Maximal serum creatinine levels were maintained from 24 to 48 h after glycerol administration, and then decreased to a level that was slightly higher than that of the control (0 h) from 72 to 120 h. Serum creatinine levels were increased in both the anisodamine and atropine groups at 24–48 h compared with all other time points; however, the levels increased more slowly in the anisodamine group compared with the atropine group. In addition, serum creatinine levels were significantly higher from 24 to 48 h in the glycerol and atropine groups compared with the anisodamine group at the corresponding time points (Fig. 3a).

Compared with 0 h, blood urea nitrogen levels increased significantly, with the maximal value at 24 h, followed by a decease at 48 h during the renal dysfunction period in all model groups (Fig. 3b). Additionally, blood urea nitrogen levels were significantly higher at 12 and 24 h in the glycerol group compared with the anisodamine group at the corresponding time points. However, blood urea nitrogen levels were not changed from 48 to 120 h in any group.

Creatine kinase levels were significantly increased at 6 h, followed by a decrease at 12 h, compared with the other time points after glycerol treatment. The changes in serum creatine kinase levels followed the same trend in the anisodamine and atropine groups as well as the glycerol group. However, the creatine kinase levels were significantly higher at 6 h in the glycerol group compared with the anisodamine and atropine groups (Fig. 3c).

### Anisodamine decreases MDA levels and increases SOD activity after glycerol-induced AKI

**Fig. 4:**
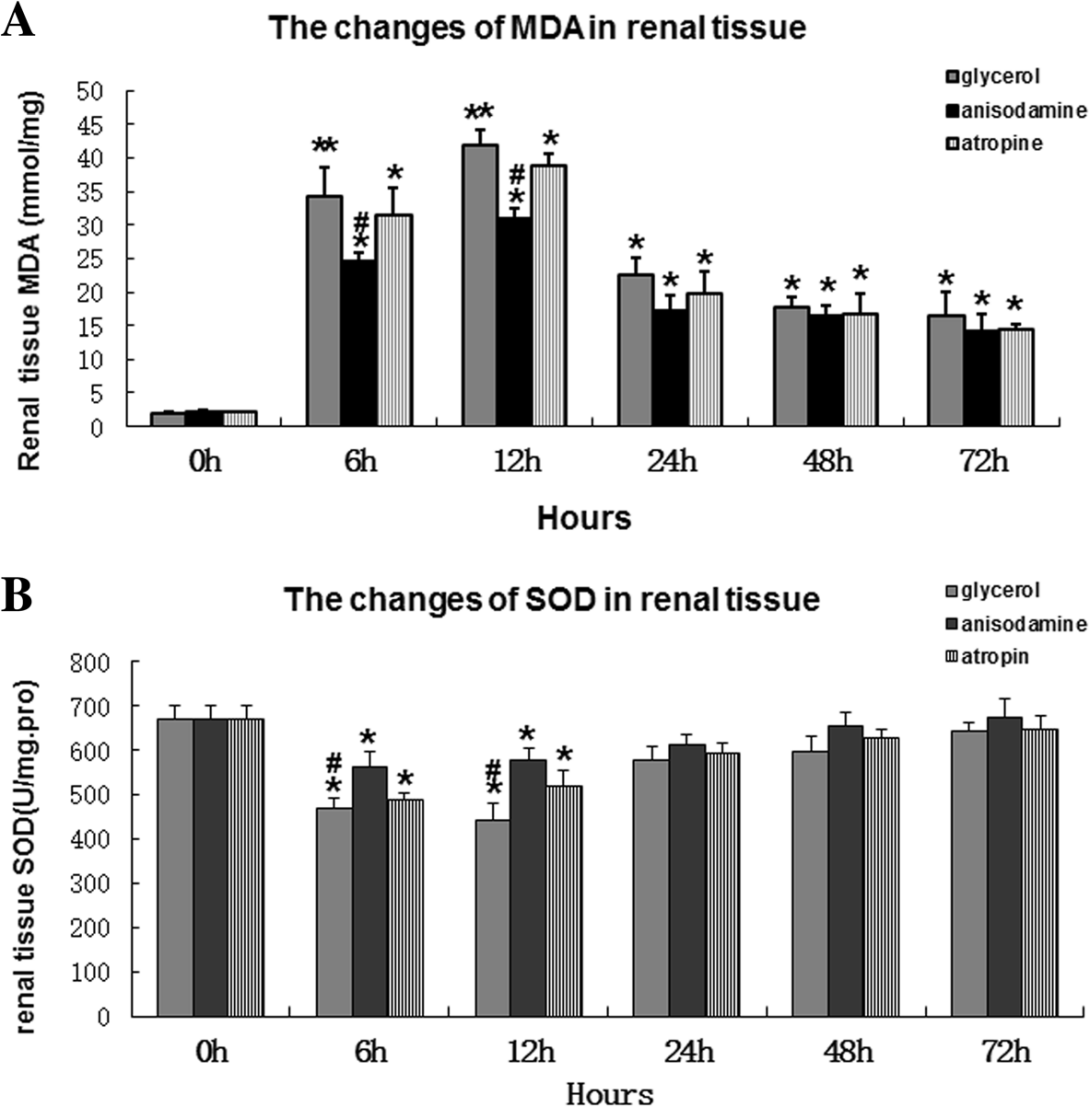
Effect of advanced administration of adnisodamine/atropine on renal ROS in rats subjected to glycerol-induced AKI. **a** tissue MDA in groups of male SD rats (*n* = 7) that were given intramuscular injections 50% glycerol (10 ml/kg), with anisodamine (1 mg/kg) and atropine (0.05 mg/kg) with intraperitoneal injection before 20 min glycerol treatment. One more group did not receive any treatment. Data are expressed as mean ± SEM (*n* = 6). #, statistically significant from respective 0 h controls. *, statistically significant from rats receiving glycerol treatment alone at the corresponding time point (*p* < 0.05). **b** tissue SOD in groups of male SD rats (*n* = 6) that were given intramuscular injections 50% glycerol (10 ml/kg), with anisodamine (1 mg/kg) and atropine (0.05 mg/kg) with intraperitoneal injection before 20 min glycerol treatment. One more group did not receive any treatment. Data are expressed as mean ± SEM (*n* = 6), statistically significant from respective 0 h controls. *, statistically significant from rats receiving glycerol treatment alone at the corresponding time point (*p* < 0.05). *P < 0.05;*P* < 0.01 versus 6, 12,24,48,72 h; #*P* < 0.05; versus the value of anisodamine group at the same time point

ROS and antioxidant defense mechanisms play an important role in the kidney [22]. SOD activity and MDA content are important indicators of antioxidant defense capacity and oxidative injury, and are useful for assessing kidney damage. As shown in Fig. 4a, MDA levels in renal tissue homogenates increased at 6 h in the different groups, peaking at 12 h, and then gradually decreased to moderate/low levels (*P* < 0.05). Additionally, MDA levels were significantly higher in the glycerol group compared with the anisodamine group at 6 and 12 h (*P* < 0.05).

SOD activity in renal tissue homogenates decreased at 6 and 12 h, and then gradually returned to normal levels (*P* < 0.05). In addition, SOD levels were significantly lower in the glycerol group compared with the anisodamine group at 6 and 12 h (*P* < 0.05) (Fig. 4b).

### Anisodamine ameliorates apoptosis/necroptosis after glycerol-induced AKI

**Fig. 5:**
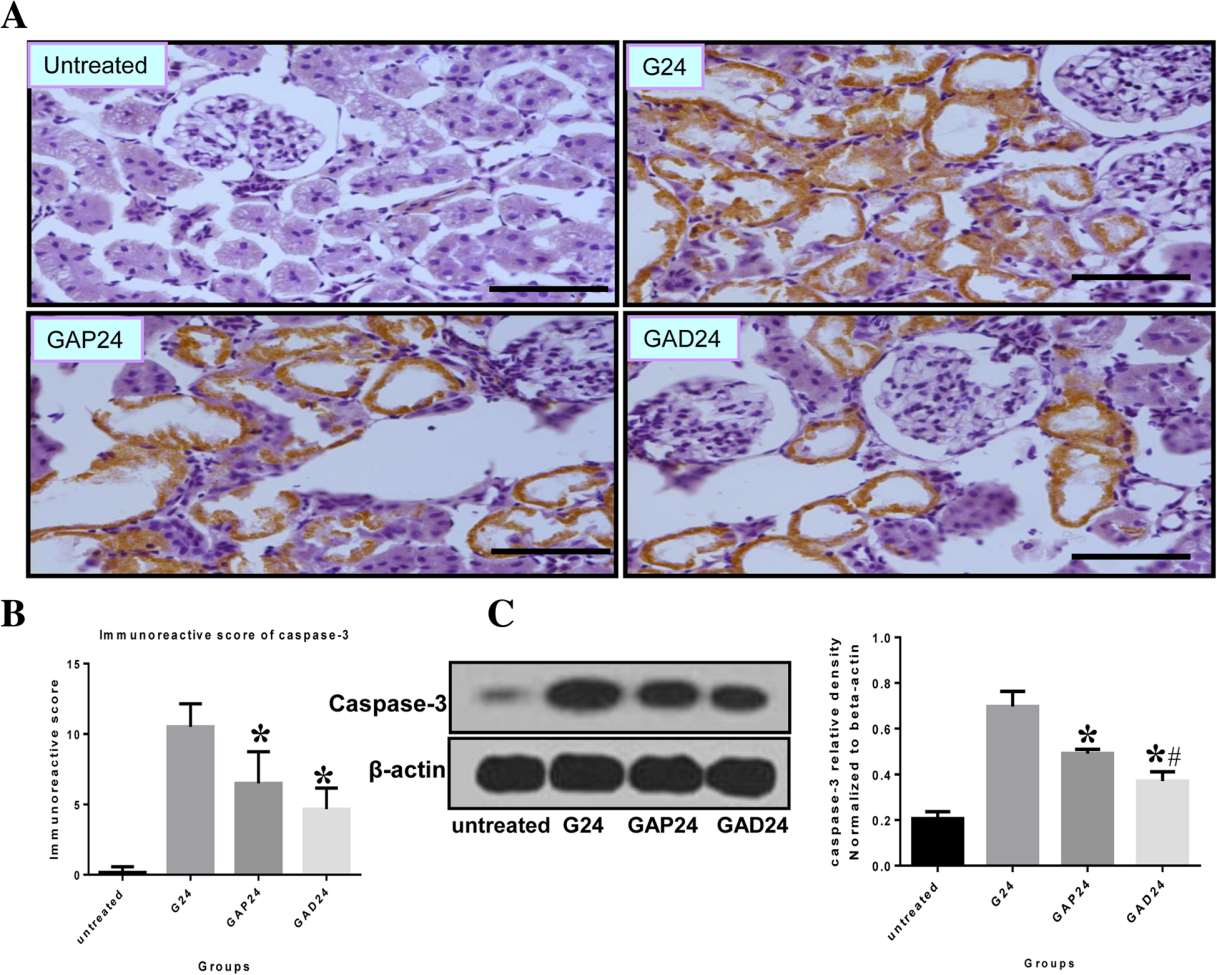
Effect of administration of adnisodamine/atropine on caspase-3 in rats subjected to glycerol-induced AKI. **a** Caspase-3 immunostaining in cross sections of rat kidney at 24 h in untreated, glycerol-treated, and adnisodamine/atropine intervention after glycerol treatment groups over the time course. Caspase-3 positive staining was observed on the proximal tubular epithelial cells and damaged tubules. U, untreated; G, glycerol treatment alone group; GAP, glycerol atropine treatment group. GAD, glycerol adnisodamine treatment group. Bars = 50 μm. Data are the mean ± SEM from three separate experiments. **b.** The statistic evaluation of immunostaining of caspase-3 (*P < 0.05 versus G24 group). **c** Total kidney tissue extracts were analyzed for caspase-3 protein levels by western blot. Data are expressed as mean ± S.E. (*n* = 3), statistically significant from respective 0 h controls. *, statistically significant from rats receiving glycerol treatment alone at the corresponding time point (*p* < 0.05)

**Fig. 6:**
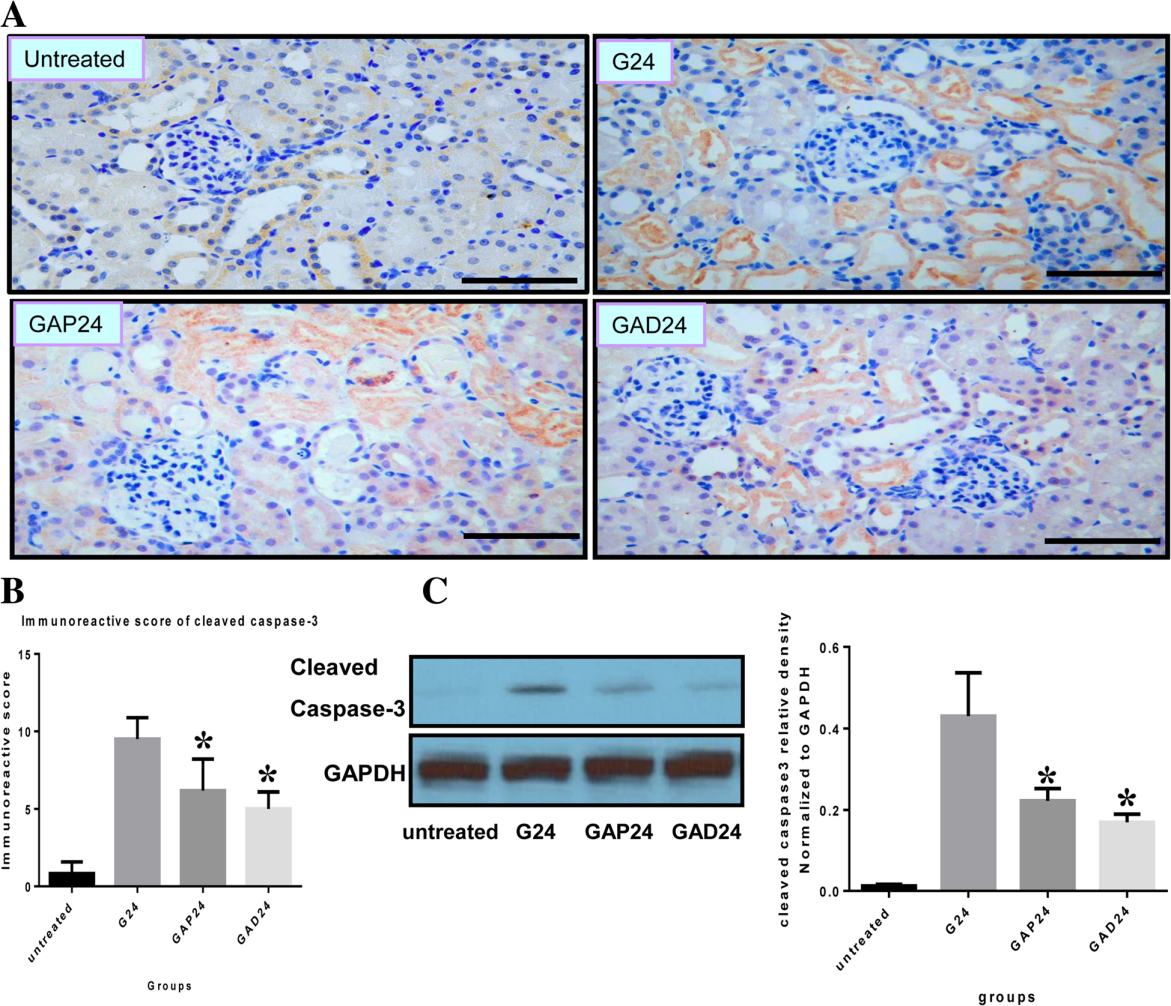
Effect of administration of adnisodamine/atropine on cleaved caspase-3 in rats subjected to glycerol-induced AKI. **a** Cleaved caspase-3 immunostaining in cross sections of rat kidney at 24 h in untreated, glycerol-treated, and adnisodamine/atropine intervention after glycerol treatment groups over the time course. Cleaved caspase-3 positive staining was observed on the proximal tubular epithelial cells and damaged tubules. U, untreated; G, glycerol treatment alone group; GAP, glycerol atropine treatment group. GAD, glycerol adnisodamine treatment group. Bars = 50 μm. Data are the mean ± SEM from three separate experiments. **b**. The statistic evaluation of immunostaining of Cleaved caspase-3 (*P < 0.05 versus G24 group)**. c**. Total kidney tissue extracts were analyzed for cleaved caspase-3 protein levels by western blot. Data are expressed as mean ± S.E. (*n* = 3), statistically significant from respective 0 h controls. *, statistically significant from rats receiving glycerol treatment alone at the corresponding time point (*p* < 0.05)

Glycerol-induced AKI is associated with caspase-3-mediated apoptosis, which results in tubular damage [11, 13]. As shown in Fig. 5a and 6a, immunohistochemical analysis revealed that caspase-3 and cleaved caspase-3 positive cells were mainly localized in the apical membranes of damaged proximal tubular epithelial cells, with desquamation and necrosis at 24 h, in kidneys with glycerol-induced tissue damage. In atropine and anisodamine-treated kidneys, a reduction in caspase-3 expression was mainly found in apical membranes of damaged proximal tubular epithelial cells, along with desquamation and necrosis, at 24 h. Moreover, only weak caspase-3 signals were observed in the anisodamine-treated kidneys compared with atropine-treated kidneys at the same time points.

We next analyzed protein levels of caspase-3 and cleaved caspase-3 in kidney tissues at 24 h. By western blotting, we observed that glycerol caused a rapid increase in caspase-3 protein levels. In comparison to the glycerol group, caspase-3 and cleaved caspase-3 expression was significantly decreased in the anisodamine and atropine groups at the same time point (Figs. 5c and 6c). In addition, levels of renal caspase-3 and cleaved caspase-3 were lower in the anisodamine group compared with the atropine group 24 h after glycerol administration.

**Fig. 7:**
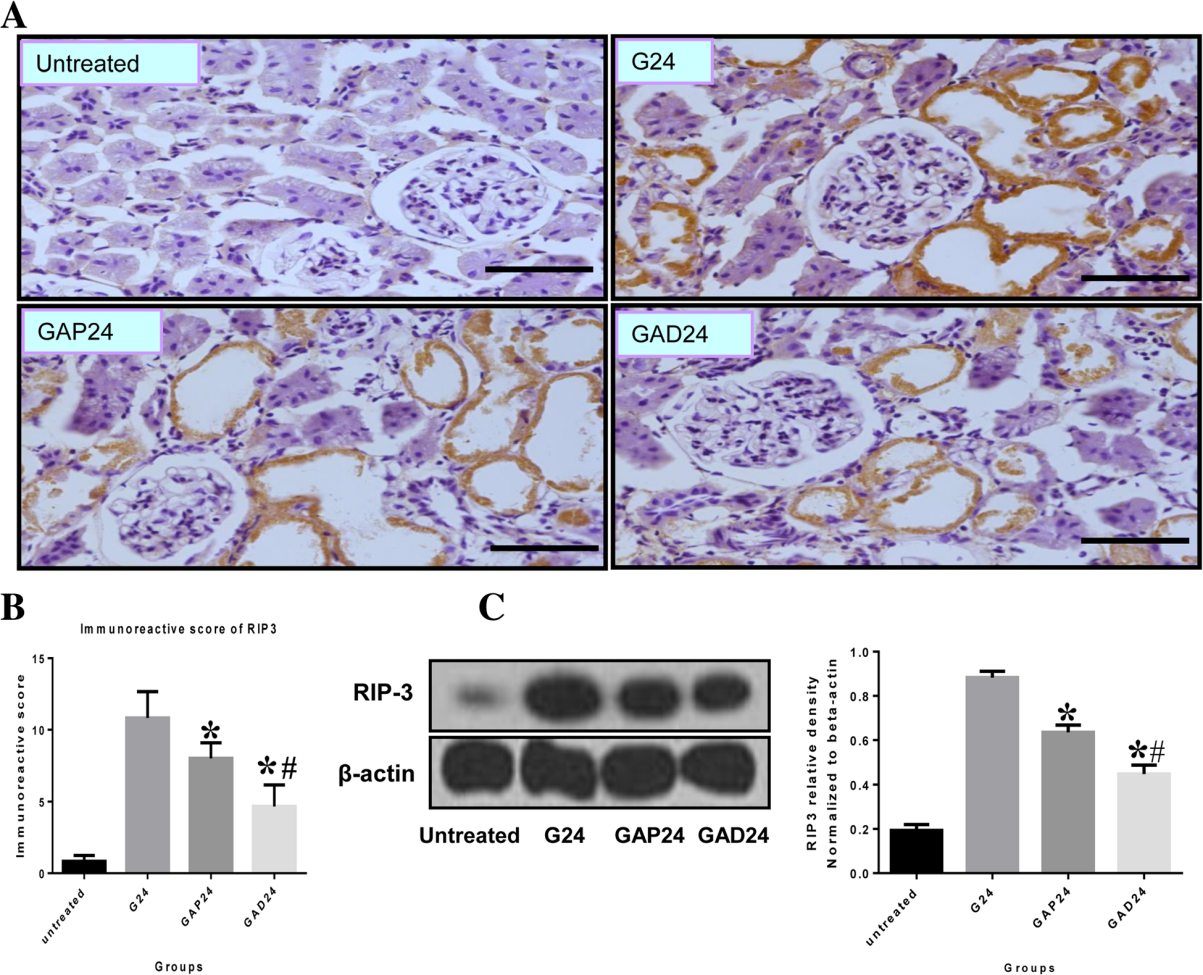
Effect of administration of adnisodamine/atropine on in rats RIP-3 subjected to glycerol-induced AKI. **a** RIP-3 immunostaining in cross sections of rat kidney at 24 h in untreated, glycerol-treated, and adnisodamine/atropine intervention after glycerol treatment groups over the time course. RIP-3 positive staining was observed on the proximal tubular epithelial cells and damaged tubules (arrows). U, untreated; G, glycerol treatment alone group; GAP, glycerol atropine treatment group. GAD, glycerol adnisodamine treatment group. Bars = 50 μm. Data are the mean ± SEM from three separate experiments. **b** The statistic evaluation of immunostaining of RIP3 (*P < 0.05 versus G24 group). **c** Total kidney tissue extracts were analyzed for RIP-3 protein levels by western blot. Data are expressed as mean ± S.E. (*n* = 3), statistically significant from respective 0 h controls. *, statistically significant from rats receiving glycerol treatment alone at the corresponding time point (*p* < 0.05)

Apoptosis and necroptosis are closely related and co-occur in photoreceptor programmed cell death [23]. We examined whether apoptosis and necroptosis co-occur in glycerol-induced cell death of proximal tubular epithelial cells. By immunohistochemical analysis, RIP3 was found to be restricted in its localization to apical membranes of damaged proximal tubular epithelial cells, along with desquamation and necrosis, at 24 h in kidneys with glycerol-induced tissue damage, consistent with the localization of caspase-3 (Fig. 7a). In atropine-treated as well as anisodamine-treated kidneys, RIP-3 signals were weaker and were mainly localized in apical membranes of damaged proximal tubular epithelial cells, with desquamation and necrosis, at 24 h. In addition, weak caspase-3 signals were observed in the anisodamine group compared with the atropine group at the same time point. RIP3 protein levels were substantially increased in kidneys with glycerol-induced damage compared with the other groups at 24 h (Fig. 7c). Levels of renal RIP3 were lower in the anisodamine group compared with the atropine group 24 h after glycerol administration.

### Anisodamine decreases leukocyte infiltration and inflammation after glycerol-induced AKI

**Fig. 8:**
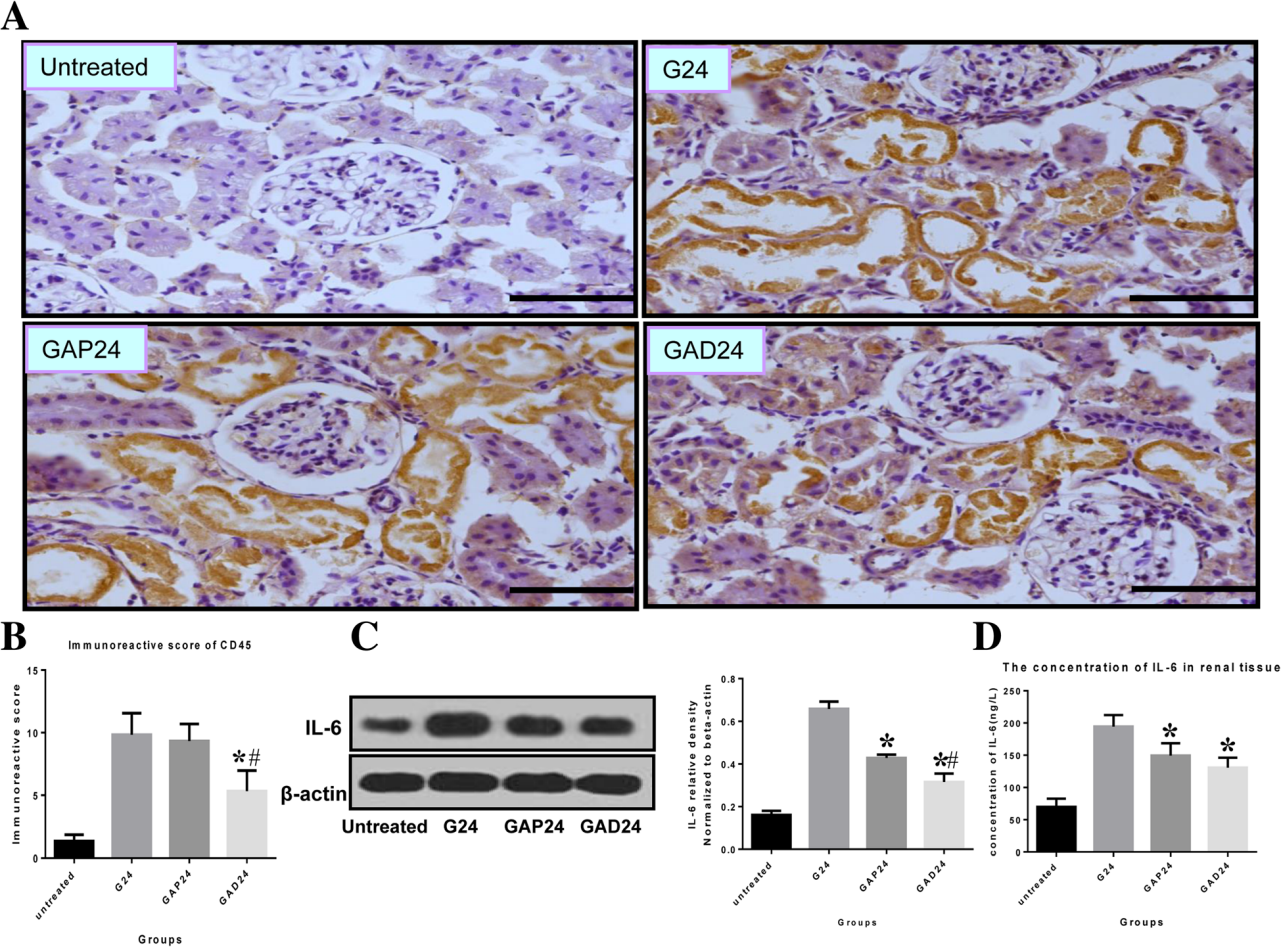
Effect of administration of adnisodamine/atropine on in rats IL-6 subjected to glycerol-induced AKI. **a** CD45 immunostaining in cross sections of rat kidney at 24 h in untreated, glycerol-treated, and adnisodamine/atropine intervention after glycerol treatment groups over the time course. CD45 positive staining was observed on the proximal tubular epithelial cells and damaged tubules (arrows). U, untreated; G, glycerol treatment alone group; GAP, glycerol atropine treatment group. GAD, glycerol adnisodamine treatment group. Bars = 50 μm. Data are the mean ± SEM from three separate experiments (**b**) The statistic evaluation of immunostaining of CD45 (*P < 0.05 versus G24 group) (**c**) Total kidney tissue extracts were analyzed for IL-6 protein levels by western blot. **d** Total kidney tissue extracts were assayed for IL-6 activity by ELISA (*P < 0.05 versus G24 group) .Data are expressed as mean ± S.E. (*n* = 3)

Many inflammatory and immune factors have been implicated in AKI [24–26], and accumulating evidence indicates that the pathophysiology of the disease is associated with inflammatory immune reactions. We performed CD45 immunostaining to detect neutrophils and monocytes to assess leukocyte infiltration. As shown in Fig. 8a, increased CD45 immunoreactivity was observed 24 h after exposure to glycerol. The immunoreactivity was diminished by anisodamine and atropine treatment at the corresponding time points. The CD45 signal was lower in the anisodamine group compared with the atropine group at the corresponding time points.

We examined IL-6 expression by western blotting and ELISA to assess the inflammatory response. Pretreatment with anisodamine and atropine markedly decreased IL-6 protein levels in the kidney of the glycerol-treated animals. The levels of renal IL-6 were lower in the anisodamine group than in the atropine group 24 h after glycerol administration (Fig. 8c, e).

### Anisodamine treatment decreases KIM-1 expression after glycerol-induced AKI

**Fig. 9:**
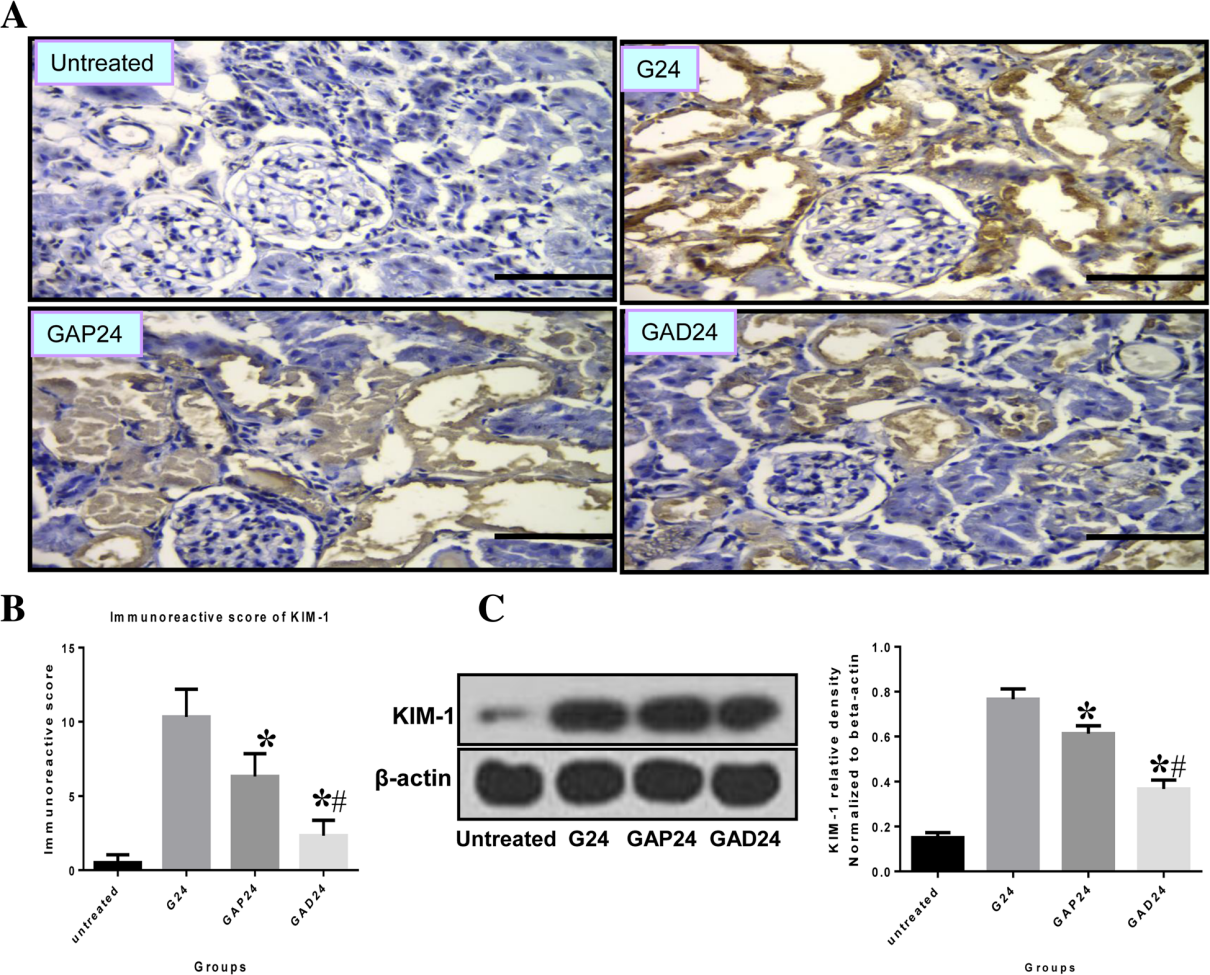
Effect of administration of adnisodamine/atropine on KIM-1 in rats subjected to glycerol-induced AKI. **a** KIM-1 immunostaining in cross sections of rat kidney at 24 h in untreated, glycerol-treated, and adnisodamine/atropine intervention after glycerol treatment groups over the time course. KIM-1 positive staining was observed on the proximal tubular epithelial cells and damaged tubules (arrows). U, untreated; G, glycerol treatment alone group; GAP, glycerol atropine treatment group. GAD, glycerol adnisodamine treatment group. Bars = 50 μm. Data are the mean ± SEM from three separate experiments. **b** The statistic evaluation of immunostaining of KIM-1 (*P < 0.05 versus G24 group). **c** Total kidney tissue extracts were analyzed for KIM-1 protein levels by western blot. Data are expressed as mean ± S.E. (*n* = 3)

KIM-1 is a sensitive biomarker of kidney injury, and was used to evaluate kidney damage and repair after AKI [27]. Immunohistochemical analysis demonstrated KIM-1 labeling in membranes of damaged proximal epithelial cells undergoing desquamation and necrosis 24 h after glycerol treatment. In atropine-treated as well as anisodamine-treated animals, KIM-1 staining was mainly localized to the apical membranes of damaged proximal tubular epithelial cells undergoing desquamation and necrosis at the corresponding time point. No significant difference in KIM-1 labeling was observed between anisodamine-treated and atropine-treated animals at 24 h (Fig. 9a). We also observed increased expression of KIM-1 in the group treated with glycerol alone at 0 to 24 h. KIM-1 protein levels were higher in the anisodamine treatment group compared with the atropine treatment group 24 h after glycerol administration (Fig. 9c).

### Anisodamine significantly decreases mortality rate in rats with glycerol-induced AKI compared with atropine

We next examined the effects of anisodamine and atropine on mortality rate after glycerol administration. The dose of 50% glycerol was increased to 15 mL/kg, which should result in death in most rats. Two independent experiments were performed to investigate the effect of anisodamine. As shown in Table 2, anisodamine significantly decreased the mortality rate in rats with glycerol-induced AKI compared with atropine (*P* < 0.05) in the two experiments. In addition, a significantly difference in mortality was observed between the anisodamine and atropine groups. No significant difference was detected between the low and high doses of atropine.

**Table 2:** Anisodamine decreased the mortality rate in rats with glycerol-induced AKI compared with atropine

		Glycerol 15 mL/kg	Anisodamine 1 mg/Kg	Atropine 0.05 mg/kg	Atropine 0.25 mg/kg
The first	Total(n)	13	10	9	7
	Living(n)	1	8	3	2
	*P*		*P1*(0.013)	*P2*(0.264)	*P3*(0.253)
	*P*			*P4*(0.049)	*P5*(0.042)
The second	Total(n)	12	9	8	8
	Living(n)	2	8	3	4
	*P*		*P*-*a*(0.01)	*P-b*(0.161)	*P*-*c*(0.603)
	*P*			*P*-*d*(0.029)	*P*-*e*(0.047)

## Discussion

In the present study, we demonstrated that anisodamine ameliorates renal damage possibly by regulating tubular leukocyte infiltration, by inhibiting apoptosis or necroptosis, and by decreasing the levels of oxidative stress. Our findings are consistent with previous studies in which oxidative stress injury, renal tubular apoptosis and systemic or local inflammation have been implicated in glycerol-induced renal dysfunction [10–12, 28]. Furthermore, anisodamine treatment was more effective than atropine treatment for glycerol-induced AKI, although they are both anti-muscarinic drugs exhibiting the usual spectrum of pharmacological effects of this drug class.

In most studies, AKI is induced by intramuscular injection of 50% glycerol (10 mL/kg) in the hind limb. This dose of glycerol is non-lethal in most rats. In comparison, 50% glycerol at 15 mL/kg can result in death. However, the cause of death was not examined in this study. The rats only showed periods of oliguria and mania-like symptoms. Treatment with anisodamine reduced the mortality rate, perhaps by improving intrarenal blood flow or by protecting against myocyte injury [1]; however, the mechanisms remain unclear.

Atropine, a centrally-acting muscarinic cholinergic receptor antagonist, may have different functions at different doses. In a study of neurons in the subfornical organ, atropine had an antagonistic action on muscarinic responses at low concentrations (0.01–1 μM), while it suppressed GABAergic synaptic transmission at high concentrations (10 μM to 1 mM) [29]. Therefore, we investigated the effect of a therapeutic dose of atropine on glycerol-induced kidney dysfunction. The high-dose group received 2 mg/kg of atropine, whereas the low-dose group received 0.05 mg/kg of atropine. We found that both doses of atropine had a modest effect on mortality rate in rats with glycerol-induced AKI, with no significant difference between the high-dose and low-dose groups.

Anisodamine also exhibits antioxidant activity, and its therapeutic effectiveness has been demonstrated in cardiac arrest and myocardial dysfunction [30, 31]. Previous studies have suggested that ROS-induced oxidative stress is an important mechanism in the initiation and maintenance of glycerol-induced AKI [30–32]. To assess the effect of anisodamine on redox status, we measured MDA levels and SOD activity in renal tissues. The increased SOD activity and decreased MDA levels suggest that anisodamine protects against early AKI by reducing ROS-induced oxidative stress and by enhancing endogenous antioxidant defense capacity.

As oxidative stress is directly involved in the pathogenesis of early AKI, it can also result in mitochondrial-related apoptosis and exacerbate renal dysfunction [32, 33]. Therefore, we measured levels of a key apoptotic protein, caspase-3/cleaved caspase-3, to assess apoptotic cell death. Although the protective effect of anisodamine against myocardial cell apoptosis has been demonstrated in pigs, its effect on apoptosis in glycerol-induced AKI remained unknown. Cells in the distal portion of the proximal tubule undergo both apoptosis and necrosis in AKI [34, 35]. Here, we found that both RIP3 and caspase-3 are localized to the membranes of damaged proximal tubular epithelial cells undergoing desquamation and necrosis, although the relationship between apoptosis and necroptosis in glycerol-induced AKI in these experiments remains unclear. After treatment with anisodamine, renal function and pathological changes were significantly improved, suggesting that necroptosis mediated by RIP3 participates in the loss of renal cells and may be an important cause of AKI.

KIM-1, a biomarker of kidney injury that is localized to damaged epithelial cells in the renal proximal tubule, was continuously expressed during the processes of kidney injury and recovery after AKI [36]. We observed that the expression of KIM-1 quickly increased (at 3 h) after the glycerol injection (data not shown), and was highly increased and sustained from 24 to 72 h. Although anisodamine and atropine both significantly decreased the expression of KIM-1 at the 24 h time point, anisodamine was much more effective in decreasing KIM-1 levels than atropine.

A pro-inflammatory response and leukocyte infiltration are typical pathophysiological characteristics of rhabdomyolysis-induced AKI, which may impair cellular functions and lead to tubular epithelial cell swelling, apoptosis, desquamation and repair [37–40]. We observed that IL-6 and CD45 levels were reduced by both anisodamine and atropine, with no significant difference between these drugs. We also found that anisodamine decreased leukocyte infiltration and protected against renal dysfunction, consistent with previous studies showing that macrophage infiltration is linked to renal dysfunction in AKI [39, 41].

In summary, we demonstrated that anisodamine promotes renal recovery in the rat model of glycerol-induced AKI. Anisodamine inhibited delayed apoptosis, and decreased inflammation and oxidative stress in renal tubular epithelial cells. Although the mechanisms underlying the nephroprotective action of anisodamine remain unclear, our findings suggest that the drug may have therapeutic potential for rhabdomyolysis-induced and other forms of AKI.

## Conclusions

We used the acute renal injury model induced by glycerol which leads to rhabdomyolysis to study the mechanism of prevention by anisodamine. Effects of the delayed administration of anisodamine on renal function and pathology in a rat model of glycerol-induced AKI were examined. We uncovered some non-M-receptor-mediated mechanisms underlying glycerol-induced AKI and described the effects of anisodamine on predictive biomarkers of AKI, signaling involved in leukocyte infiltration and inflammation, oxidative stress and apoptosis. Retreatment by anisodamine ameliorated renal dysfunction in glycerol-induced rhabdomyolysis by inhibiting oxidative stress, inflammatory response and apoptosis (necrosis). Further studies are needed to prospectively explore mechanism of apoptosis of renal epithelial cells presumably involved in transport of membrane protein such as megalin or lysosome-related apoptosis due to excessive intake of myohemoglobin.

## Data Availability

All data generated or analysed during this study are included in this published article.

## Abbreviations

AKI: Acute kidney injury
*BUN*: Blood urea nitrogen
CK: Creatine kinase
IL-6: Interleukin 6
KIM-1: Kidney injury molecular-1
MDA: Malondialdehyde
Scr: Serum creatinine
SOD: Superoxide dismutase

## References

[1] Holt SG, Moore KP (2001). Pathogenesis and treatment of renal dysfunction in rhabdomyolysis. Intensive Care Med.

[2] Melli G, Chaudhry V, Cornblath DR (2005). Rhabdomyolysis:an evaluation of 475 hospitalized patients. Medicine (Baltimore).

[3] Mousleh R, Al Laham S, Al-Manadili A (2018). The preventive role of pioglitazone in glycerol-induced acute kidney injury in rats during two different treatment periods. Iran J Med Sci.

[4] Gburek J, Birn H, Verroust PJ, Goj B, Jacobsen C, Moestrup SK, Willnow TE, Christensen EI (2003). Renal uptake of myoglobin is mediated bythe endocytic receptors megalin and cubilin. Am J Physiol Renal Physiol..

[5] Reeder BJ, Wilson MT (2005). Hemoglobin and myoglobin assoeiated oxidative stress: from moleular mehanisms to disease states. Curr Med Chem.

[6] Zager RA, Burkhart K (1997). Myoglobin toxicity in proximal human kidney cells: roles of Fe, Ca2+, H_2_O_2_, and terminal mitochondrial electron transport. Kidney Int.

[7] Wu J, Pan X, Fu H, Zheng Y, Dai Y, Yin Y, Chen Q, Hao Q, Bao D, Hou D (2017). Effect of curcumin on glycerol-induced acute kidney injury in rats. Sci Rep.

[8] Kim JH, Lee SS, Jung MH, Yeo HD, Kim HJ, Yang JI, Roh GS, Chang SH, Park DJ (2010). N-acetylcysteine attenuates glycerol-induced acute kidney injury by regulating MAPKs and Bcl-2 family proteins. Nephrol Dial Transplant.

[9] Wei Q, Hill WD, Su Y, Huang S, Dong Z (2011). Heme oxygenase-1 induction contributes to renoprotection by G-CSF during rhabdomyolysis-associated acute kidney injury. Am J Physiol Renal Physiol.

[10] Al Asmari AK, Al Sadoon KT, Obaid AA, Yesunayagam D, Tariq M (2017). Protective effect of quinacrine against glycerol-induced acute kidney injury in rats. BMC Nephrol.

[11] Homsi E, de Brito SM, Janino P (2010). Silymarin exacerbates p53-mediated tubular apoptosis in glycerol-induced acute kidney injury in rats. Ren Fail.

[12] Wang YD, Zhang L, Cai GY, Zhang XG, Lv Y, Hong Q, Shi SZ, Yin Z, Liu XF, Chen XM (2011). Fasudil ameliorates rhabdomyolysis-induced acute kidney injury via inhibition of apoptosis. Ren Fail.

[13] Korrapati MC, Shaner BE, Schnellmann RG (2012). Recovery from glycerol-induced acute kidney injury is accelerated by suramin. J Pharmacol Exp Ther.

[14] Xiu RJ, Hammerschmidt DE, Coppo PA, Jacob HS (1982). Anisodamine inhibits thromboxane synthesis, granulocyte aggregation, and platelet aggregation. A possible mechanism for its efficacy in bacteremic shock. JAMA.

[15] Yao BJ, He XQ, Lin YH, Dai WJ (2018). Cardioprotective effects of anisodamine against myocardial ischemia/reperfusion injury through the inhibition of oxidative stress, inflammation and apoptosis. Mol Med Rep.

[16] You QH, Zhang D, Niu CC, Zhu ZM, Wang N, Yue Y, Sun GY (2014). Expression of IL-17A and IL-17F in lipopolysaccharide-induced acute lung injury and the counteraction of anisodamine or methylprednisolone. Cytokine.

[17] Peng Y, Fu X, Li W, Geng W, Xing K, Ru L, Sun J, Zhao Y (2014). Effect of intracoronary anisodamine and diltiazem administration during primary percutaneous coronary intervention in acute myocardial infarction. Coron Artery Dis.

[18] Poupko JM, Baskin SI, Moore E (2007). The pharmacological properties of anisodamine. J Appl Toxicol.

[19] Zou AP, Parekh N, Steinhausen M (1990). Effect of anisodamine on the microcirculation of the hydronephrotic kidney of rats. Int J Microcirc Clin Exp.

[20] Abul-Ezz SR, Walker PD, Shah SV (1991). Role of glutathione in an animal model of myoglobinuric acute renal failure. Proc Natl Acad Sci U S A.

[21] Wang Z, Shah SV, Liu H, Baliga R (2014). Inhibition of cytochrome P450 2E1 and activation of transcription factor Nrf2 are renoprotective in myoglobinuric acute kidney injury. International Society of Nephrology Kidney Int.

[22] Turkmen K (2016). Inflammation, oxidative stress, apoptosis, and autophagy in diabetes mellitus and diabetic kidney disease: the four horsemen of the apocalypse. Int Urol Nephrol.

[23] Trichonas G, Murakami Y, Thanos A, Morizane Y, Kayama M, Debouck CM, Hisatomi T, Miller JW, Vavvas DG (2010). Receptor interacting protein kinases mediate retinal detachment-induced photoreceptor necrosis and compensate for inhibition of apoptosis. Proc Natl Acad Sci U S A.

[24] Jang HR, Rabb H (2015). Immune cells in experimental acute kidney injury. Nat Rev Nephrol.

[25] Karwasra R, Kalra P, Gupta YK, Saini D, Kumar A, Singh S (2016). Antioxidant and anti-inflammatory potential of pomegranate rind extract to ameliorate cisplatin-induced acute kidney injury. Food Funct.

[26] Ruiz-Andres O, Suarez-Alvarez B, Sánchez-Ramos C, Monsalve M, Sanchez-Niño MD, Ruiz-Ortega M, Egido J, Ortiz A, Sanz AB (2016). The inflammatory cytokine TWEAK decreases PGC-1α expression and mitochondrial function in acute kidney injury. Kidney Int.

[27] Huang Y, Don-Wauchope AC (2011). The clinical utility of kidney injury molecule 1 in the prediction, diagnosis and prognosis of acute kidney injury: a systematic review. Inflamm Allergy Drug Targets.

[28] Rosenberger C, Goldfarb M, Shina A, Bachmann S, Frei U, Eckardt KU, Schrader T, Rosen S, Heyman SN (2008). Evidence for sustained renal hypoxia and transient hypoxia adaptation in experimental rhabdomyolysis-induced acute kidney injury. Nephrol Dial Transplant.

[29] Xu SH, Ono K, Honda E, Inenaga K (2002). Noncholinergic actions of atropine on GABA ergic synaptic transmission in the subfornical organ of rat slice preparations. Toxicol Appl Pharmacol.

[30] Palipoch S (2013). A review of oxidative stress in acute kidney injury: protective role of medicinal plants-derived antioxidants. Afr J Tradit Complement Altern Med.

[31] Arora S, Kaur T, Kaur A, Singh AP (2014). Glycine aggravates ischemia reperfusion-induced acute kidney injury through n-methyl-d-aspartate receptor activation in rats. Mol Cell Biochem.

[32] Guo SX, Zhou HL, Huang CL, You CG, Fang Q, Wu P, Wang XG, Han CM (2015). Astaxanthin attenuates early acute kidney injury following severe burns in rats by ameliorating oxidative stress and mitochondrial-related apoptosis. Mar Drugs.

[33] Wagener FA, Dekker D, Berden JH, Scharstuhl A, van der Vlag J (2009). The role of reactive oxygen species in apoptosis of the diabetic kidney. Apoptosis.

[34] Linkermann A, Himmerkus N, Rölver L, Keyser KA, Steen P, Bräsen JH, Bleich M, Kunzendorf U, Krautwald S (2011). Renal tubular Fas ligand mediates fratricide in cisplatin-induced acute kidney failure. Kidney Int.

[35] Safirstein RL (2011). Am I my brother’s keeper?: fratricide in the kidney. Kidney Int.

[36] Yin C, Wang N (2016). Kidney injury molecule-1 in kidney disease. Ren Fail.

[37] Luo ZY, Tang Y, You JI, Luo H (1992). Protective effect of anisodamine on cultured bovine pulmonary endothelial cell injury induced by oxygen-free radicals. Arch Surg.

[38] Zhao T, Li DJ, Liu C, Su DF, Shen FM, Li CH, Zhang X, Ge XL, Huang X, Zhang AQ, Gu WQ (2011). Beneficial effects of anisodamine in shock involved cholinergic anti-inflammatory pathway. Front Pharmacol.

[39] Li CH, Zhang X, Ge XL, Huang X, Zhang AQ, Gu WQ (2014). Effects of combined anisodamine and neostigmine treatment on the inflammatory response and liver regeneration of obstructive jaundice rats after hepatectomy. Biomed Res Int.

[40] Qian J, Zhang JM, Lin LL, Dong WZ, Cheng YQ, Su DF, Liu AJ, Wei Z, Bing Z, Jiu J, Xue Y (2015). A combination of neostigmine and anisodamine protects against ischemic stroke by activating α7nAChR. Int J Stroke.

[41] Palmieri T, Lavrentieva A, Greenhalgh DG (2010). Acute kidney injury in critically ill burn patients. Risk factors, progression and impact on mortality. Burns.

